# Proof-of-concept study of the efficacy of a microbiota-directed complementary food formulation (MDCF) for treating moderate acute malnutrition

**DOI:** 10.1186/s12889-020-8330-8

**Published:** 2020-02-17

**Authors:** Ishita Mostafa, Naila Nurun Nahar, Md. Munirul Islam, Sayeeda Huq, Mahfuz Mustafa, Michael Barratt, Jeffrey I. Gordon, Tahmeed Ahmed

**Affiliations:** 1 Nutrition and Clinical Services Division (NCSD), International Centre for Diarrhoeal Disease Research, Bangladesh (icddr, b), 68, Shaheed Tajuddin Ahmed Sarani Mohakhali, Dhaka, 1212 Bangladesh; 2 The Edison Family Center for Genome Sciences and Systems Biology, Washington University School of Medicine, St. Louis, MO USA; 3 Center for Gut Microbiome and Nutrition Research, Washington University School of Medicine, St. Louis, MO USA; 4 James P. Grant School of Public Health, BRAC University, Mohakhali, Dhaka, 1212 Bangladesh; 5 Department of Global Health, University of Washington, Seattle, WA USA

**Keywords:** Moderate acute malnutrition (MAM), Severe acute malnutrition (SAM), Microbiota Directed Complementary Food (MDCF), Ready to use Supplementary Food (RUSF)

## Abstract

**Background:**

Childhood undernutrition remains a significant global health challenge accounting for over half of all under 5 child mortality. Moderate acute malnutrition (MAM), which leads to wasting [weight-for-length z-scores (WLZ) between − 2 and − 3], affects 33 million children under 5 globally and more than 2 million in Bangladesh alone. We have previously reported that acute malnutrition in this population is associated with gut microbiota immaturity, and in a small, 1-month pre-proof-of-concept (POC) study demonstrated that a microbiota-directed complementary food formulation (MDCF-2) was able to repair this immaturity, promote weight gain and increase plasma biomarkers and mediators of healthy growth. Here we describe the design controlled feeding study that tests whether MDCF-2 exhibits superior efficacy (ponderal growth, host biomarkers of a biological state) than a conventional Ready-to-use Supplementary Food (RUSF) in children with MAM over intervention period of 3 months.

**Methods:**

Two separate cohorts of 12–18-month-old children will be enrolled: 124 with primary MAM, and 124 with MAM after having been treated for severe acute malnutrition (post-SAM MAM). We have established several field sites in an urban slum located in the Mirpur district of Dhaka, Bangladesh and at a rural site, Kurigram in the north of Bangladesh. The two groups of children receiving MDCF-2 and RUSF will be compared at baseline (pre-intervention), after 1 month, at the end of intervention (3 months), 1 month after cessation of intervention, and every 6 months thereafter for 4 years.

**Discussion:**

This study will determine whether daily, controlled administration of MDCF-2 for 3 months provides superior improvements in weight gain, microbiota repair, and elevated levels of key plasma biomarkers/mediators of healthy growth compared to the control RUSF formulation. The pathogenesis of MAM is poorly defined and there are currently no WHO-approved treatments; results from the current study of children with primary MAM and post-SAM MAM will shed light on the effects of the gut microbiota on childhood growth/development and will provide a knowledge base that may help improve complementary feeding practices.

**Trial registration:**

The primary MAM and post-SAM MAM trials are registered in Clintrials.gov (NCT04015999 and NCT04015986, registered on July 11, 2019, retrospectively registered).

## Background

Moderate acute malnutrition (MAM), a major global health problem, is defined as wasting (weight-for-length z-scores between <− 2 and − 3 compared to WHO Child Growth Standards) and/or mid-upper-arm circumference (MUAC) greater or equal to 115 mm and less than 125 mm. According to the 2017 Global Nutrition Report, worldwide 52 million children under 5 years of age (8%) were acutely malnourished while stunting affected 23% or 155 million [1]. Bangladesh has one of the highest burdens of childhood undernutrition in the world. According to the 2014 Bangladesh Demographic Health Survey (BDHS), among children under 5, 36% were stunted, 12% had severe stunting (LAZ < -3) [2] and 15% were wasted (WLZ < -2), with more than 2 million classified as suffering from MAM, and 450,000 having severe acute malnutrition (SAM, WLZ < -3) [3]. Malnutrition costs Bangladesh an estimated 1 billion USD a year [4].

In our previous studies [5, 6], we defined a normal program of gut microbial community development in healthy members of a Bangladeshi birth cohort who had provided fecal samples monthly for the first 60 months of life. This program of community assembly is described by changes in the abundances of a group of 15 bacterial strains that together form a network of covarying organisms (an ‘ecogroup’) and is completed by the end of the second postnatal year. The same program is shared by healthy members of birth cohorts residing in other low- and middle-income countries [6]. Using the 15 ecogroup taxa, we found that Bangladeshi children with MAM and SAM have impaired gut community development; this perturbation is worse in children with MAM compared to SAM and is not repaired by currently available nutritional interventions, which are not designed based on knowledge and/or consideration of gut microbial community development [6].

Recently, we have designed prototypes for nutritional interventions that are composed of locally available, affordable, culturally acceptable complementary foods commonly consumed in Bangladesh. Preclinical studies using gnotobiotic mice and piglets colonized with members of the gut microbiota from Bangladeshi children with acute malnutrition revealed that these formulations contain nutrients that increase the representation and expressed beneficial functions of growth-promote gut bacterial strains that are underrepresented in the microbiota of affected children [5]. Several of these microbiota-directed complementary food (MDCF) formulations were subsequently tested in a pre-POC study involving 12–18-month-old Bangladeshi children with MAM living in an urban slum (Mirpur) located in a district of the nation’s capital city [5]. This 1-month long, four-arm, controlled feeding study tested three MDCFs and a commonly used, rice-lentil-based, ready-to-use supplementary food (RUSF) that was not designed with the intention of changing gut microbial community structure or function. One of the MDCFs, MDCF-2, was distinguished from the other formulations based on its superior ability to (i) repair the microbiota of children with MAM to a configuration that resembled that of healthy individuals living in the same community, and (ii) change the levels of multiple plasma proteins involved in mediating various aspects of metabolism, bone growth, immune function and neurodevelopment towards a healthy state [5, 6]. These results support the notion that repair of impaired gut microbial community development could represent a new therapeutic concept for restoring healthy growth.

The pre-POC study involved small numbers of children (15–17/arm) and the 1-month long intervention was too brief to determine whether a substantial and durable repair of the microbiota, and the changes in the plasma proteome, would be accompanied by substantial and sustained changes in ponderal and linear growth. Here, we describe our protocols for POC trials designed to assess whether compared to RUSF, administration of MDCF-2 to larger numbers of children with MAM for longer periods of time will produce durable changes in the configurations of their gut communities, their plasma proteomes, anthropometric indicators of their growth, and their morbidities. Two cohorts of 12–18-month-old Bangladeshi children will be enrolled; one with primary MAM and the other with MAM after having been treated for severe acute malnutrition (post-SAM MAM). In each cohort, children will be randomized to one of two treatment arms; members of one arm will receive MDCF-2 while members of the control arm will receive a standard RUSF. Primary clinical outcome measures will be the rate of weight gain and the change in WLZ over the intervention period. Biospecimens (blood, feces and urine) collected before, during and after the intervention will be analysed to determine the extent of gut microbiota repair and the degree of improvement in levels of plasma biomarkers and mediators of healthy growth.

### Aim and objectives

To determine whether daily, controlled administration of MDCF-2 for 3 months provides superior improvements in weight gain, microbiota repair, and elevated levels of key plasma biomarkers/mediators of healthy growth compared to the control RUSF formulation. The sustainability of microbiota repair, growth outcomes and physiologic state will be assessed over a period of 4 years through collection and analysis of fecal and blood samples every 6 months.

## Methods

### Study design

**Fig. 1:**
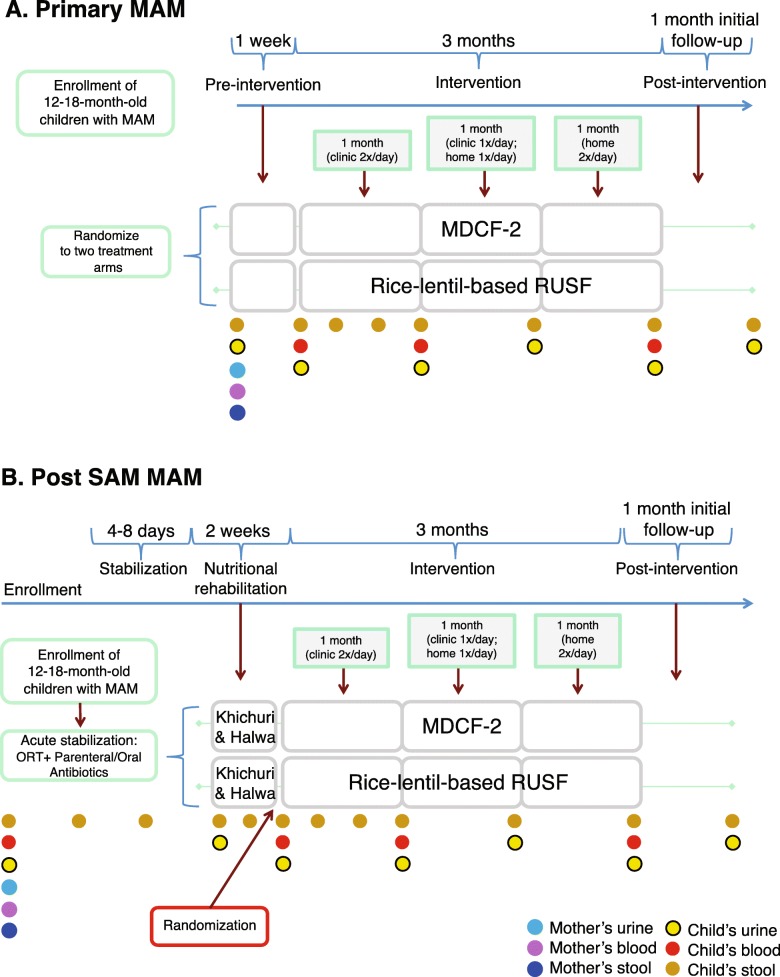
Design of controlled feeding study, including time points where anthropometric data will be collected and biospecimens obtained. **a** Trial of children presenting with primary MAM. **b** Trial of children who present with SAM

Fig. 1 describes the designs of the two open-label, randomized control trials.

Enrollment began on November, 2018 and it is expected to continue until July, 2020. Eligible participants are 12–18-month-old children of either sex with MAM (WLZ < -2 to − 3; Study 1) or SAM (WLZ < -3; Study 2). Children will be screened and enrolled through household surveys by Field Research Assistants (FRAs) following pre-specified inclusion criteria (see Table 1 for inclusion/exclusion criteria). For those who meet the enrollment criteria, a full description of the purpose of the study will be provided, including the number and type of biological samples to be collected. Parents/legal guardians who are willing to participate will provide a signed informed consent statement that is witnessed and also signed by the investigator.

**Table 1:** inclusion/ exclusion criteria of the study participants

Inclusion	
Parent(s) willing to sign consent form; the informed consent document will explicitly request permission to use the collected fecal samples for future studies, including but not limited to culturing component bacterial strains	
Child age 12–18 months and no longer exclusively breast fed	
WLZ (<−2 to −3) without bilateral pedal edema at the time of randomization	
Parent(s) willing to bring the child to the feeding center twice daily for the first 4 weeks of nutritional therapy, once daily for the second 4 weeks, and provide feeding once daily at home for the second 4 weeks, and twice daily at home for the final 4 weeks	
Exclusion	
Medical conditions: Children with tuberculosis (diagnosis based on WHO 2014 guidelines which have been incorporated in the national TB control guidelines of Bangladesh). The guidelines depend upon the following five diagnostic principles (three out of five should be positive):	
-Specific symptoms of TB	
-Specific signs of TB	
-Chest X-ray	
-Mantoux test	
-History of contact	
Any congenital/acquired disorder affecting growth, i.e., known case of trisomy-21 or cerebral palsy; children on an exclusion diet for the treatment of persistent diarrhoea; having known history of soy, peanut or milk protein allergy	
Severe anemia (< 8 mg/dl)	
Antibiotic use within the last 15 days for Primary MAM participants	
Receiving concurrent treatment for another condition	
Failure to obtain informed written consent from parents/guardians	

Participants’ guardian/ caregiver who will provide written informed consent and participants who satisfy the inclusion/exclusion criteria will be randomized to one of the two treatment arms (MDCF-2, RUSF) using the permuted block randomization method with concealment to ensure that the allocation is not made before obtaining consent from parents/caregivers to participate in the study. A random allocation sequence will be generated using a computerized random allocation system for permuted block randomization to ensure comparable allocation number at a certain equally spaced points in the sequence of participant assignment. Parallel type of randomization will be used. Blocks of 2 and 4 will be constructed to reduce the predictability. Random assignment will be prepared in advance by an independent researcher from icddr b who has no involvement in the trial. Study staffs are blinded to random assignments.

### Sample size calculation

Primary outcome measures will include rate of weight gain, change in weight and length; change in WLZ, change in MUAC, change in LAZ, morbidity, extent of repair of gut microbial community immaturity, and changes in the levels of key plasma biomarkers of host physiological state. We plan to include 124 participants (*n* = 62/treatment arm) for the primary MAM trial and for the post-SAM MAM trial. Recruitment of 124 participants will give 80% power at 5% significance level based on the change in WLZ scores of children from the recently completed pre-POC trial of different MDCFs. In the pre-POC trial of different MDCFs, the baseline WLZ score of children who received MDCF 2 was − 2.2 and after 1 month of supplementation was − 1.7. If we consider the WLZ − 2 at baseline and − 1.7 at end line, pooled standard deviation as 0.53, then the sample size would be 49 in each arm at 80% power and 5% level of significance. With 20% attrition, 62 enrolled children will be needed for each arm in each of the two trials.

### Interventions

We have established several field sites in an urban slum (Mirpur district of Dhaka). The Kurigram field site has been established in collaboration with the Terre des Hommes NGO. The post-SAM MAM study will enroll participants living in both Mirpur and Kurigram while the MAM study will be conducted in Mirpur. We have established a Food Processing Laboratory at the Mirpur and Kurigram field sites in order to freshly prepare the MDCF-2 and RUSF formulations each day prior to their distribution on that day to study participants.

Every child will be offered 25 g of the diet twice daily at the feeding center for the first 4 weeks. In the following month, the child will be offered 25 g of the diet at the feeding center and additional 25 g will be provided in a clean container to feed at home. In the third month, two separate containers containing 25 g diet will be provided every day to each enrolled child at participant’s home. Feeding information will be collected from mothers and amount consumed is determined from the weight of supplement remaining at the end of each feeding session.

In the stabilization phase of the post-SAM MAM trial, each child will be managed according to WHO/icddr,b guidelines [7]. This protocol includes programmed feeding, micronutrient supplementation, judicious rehydration, broad spectrum antibiotic treatment and prompt recognition and management of complications. Upon recovery from the stabilization phase, the child will receive a standard of care dietary protocol based upon the local diets Halwa and Khichuri. Upon graduation from SAM to MAM (edema-free WLZ < -2), children will be randomly assigned to one of the two arms.

The schedule for collection of anthropometric data and biological samples are noted in Fig. 1a, b. Children will be seen 1 month after cessation of treat for anthropometry and collection of fecal, blood and urine specimens and subsequently every 6 months for up to 4 years.

### Production of MDCF-2 and RUSF, and quality control measures

RUSF is composed of rice, lentil, sugar, soybean oil, and skimmed milk powder mixed with vitamin mineral premix. MDCF-2 is composed of chickpea flour, peanut flour, soy flour, green banana, sugar, soybean oil and vitamin-mineral premix (Table 2). Both study diets will be prepared in the icddr,b Food Processing Laboratory or nutrition centre established at each site. MDCF-2 is similar in energy density and micronutrient content to ready-to-use supplementary foods used for treatment of MAM in Bangladesh and other countries; it meets all safety and other WHO requirements for a complementary/supplementary food for 12–18-month-old children with MAM. Organoleptic acceptability was confirmed previously in the pre-POC study. Table 2 provides details about the composition, nutritional content of MDCF-2 and RUSF.

**Table 2:** Composition of the study interventions

	MDCF 2 (g/100 g)	RUSF (g/100 g)
Components	Chickpea flour (10)	Rice (18.9)
	Peanut flour (10)	Lentil (21.5)
	Soybean flour (8)	Skimmed milk powder (10.5)
	Green banana (19)	–
	Sugar (29.8)	Sugar (17)
	Soybean oil (20)	Soybean oil (29)
	Micronutrient mix (3.14)	Micronutrient mix (3.14)
Protein	11.6	10.2
Fat	20.8	29.5
Carbohydrate	46.2	48.8
Fiber	4.5	4.7
Protein-Energy ratio	11.4	8.2
Fat-Energy ratio	46.0	53.6
Total Calories (per 100 g)	406.8	494.6

We will purchase all raw ingredients for the diets from a single local market in Dhaka; these ingredients will be used to formulate the diets at both field sites. The icddr,b Food Safety Laboratory will routinely culture random samples of the prepared diets on (Luria-Bertani, Mannitol Egg Yolk Polymyxin, Tryptic Soy Broth Agar) media to quantify viable bacteria (including tests for *Escherichia coli, Bacillus cereus, Staphylococci*), yeast and other fungi. Nutritional composition will be confirmed at the Institute of Nutrition, Mahidol University, Thailand using standard procedures.

### Anthropometry, data collection tools and quality control measures

Body weight will be measured to the nearest 1 g using the Dual-Purpose Baby Scale (Seca, West Midlands, UK). Mid upper arm circumference (MUAC) will be quantified with a MUAC tape. Standing height will be determined to the nearest 0.1 cm using a Stadiometer, and supine length will be defined to the nearest 0.1 cm using an Infantometer (Seca). All scales will be calibrated daily. At each assessment, 3 consecutive measurements will be taken and the average value will be recorded. Interrater consistency (kappa) will be determined at regular intervals [8].

### Collection, preparation and archiving of biological samples

Biospecimens will be collected before, during and after the intervention at the time points indicated in Fig. 1a, b. Venous blood will be collected in EDTA containing tubes from children and their mothers (2 mL and 5 mL, respectively). Plasma will be recovered after centrifugation, aliquoted into cryovials and the vials will be stored at -80 °C. After cleaning the genital area with alcohol pads, 2 mL of urine will be collected into a collection bag from children and 5 mL from their mothers. Following centrifugation, aliquots will be maintained at -80 °C in cryovials. Fecal samples will be collected in a sterile container, transferred by the field worker within 20 min of defecation into sterile 2 mL cryovials and immediately placed in Taylor Wharton CX300 dry cryoshippers that are pre-charged with liquid N2. Upon return to the laboratory, these vials will be stored at -80^o^ C.

### Data management and storage

Procedures for data management at icddr,b have been previously described [9]. An Excel-based program will be used for scheduling data and sample collection from each participant. Feeding information, including the total amount of MDCF-2 and RUSF consumed at each treatment session, and responses to a food frequency questionnaire, will be collected by the field research supervisors. Barcoded labels are used for all laboratory specimens. Unique codes are provided for each of study sites. All reports, study data collection and administrative forms are coded to maintain study participant confidentiality and for future reference. All study-related documents are kept in locked cabinets in locked rooms with restricted access.

### Data analysis plan

The two groups of children receiving MDCF-2 and RUSF in each trial will be compared at baseline and at different time points as shown Fig. 1a, b. The clinical outcome variables will include rate of weight gain, anthropometric indices, and morbidity. We will perform t-test, Mann-Whitney test, Chi-Square test and Fisher’s exact test to compare the primary clinical outcome variables between the intervention arms. Multivariable linear regression will be employed to assess the role of the nutritional interventions to the anthropometric outcome. The data analyst will be blinded to the intervention arms.

Serially collected biospecimens will be analysed for (ii) changes in the plasma proteome (characterized by quantitative aptamer-based measurements of 5000 different proteins including those that are biomarkers and mediators of bone growth, metabolism, immune function, neurodevelopment and other aspects of physiologic status), fecal, plasma and urinary metabolomes (utilizing targeted and non-targeted mass spectrometry, including the products of metabolism carbohydrate components of the diet and of host metabolic health), (iii) repair of the gut microbiota (V4-16S rDNA amplicon sequencing to identify the abundances of ecogroup bacterial taxa and qPCR assays of the levels/diversity of common enteropathogens in fecal samples) and microbiome (shotgun sequencing of fecal DNA samples to characterize changes in the representation of microbial genes including those involved in the metabolism of various nutrients). A variety of computational tools, including those that allow for feature reduction [6, 10], will be applied to these multi-omic datasets to identify significant correlations between components of the microbiota/microbiome, proteome, metabolome and clinical parameters.

## Discussion

This study is a controlled feeding study with daily monitoring of consumption of the nutritional supplements and comprehensive assessments of feeding histories and morbidities. The pathogenesis of MAM is poorly defined and there are currently no WHO-approved treatments; results from the current study of children with primary MAM and post-SAM MAM will shed light on the effects of the gut microbiota on childhood growth/development and will provide a knowledge base that may help improve complementary feeding practices. The ability to comprehensively characterize the biological state of children with MAM by quantitative measurements of 5000 plasma proteins may define different subsets of children within the coarser classification of MAM, and will help determine the relationship between treatment effects on the levels of various mediators of growth and observed growth outcomes. The 3-month period of intervention and subsequent 4-year follow up permits assessment of the durability of effects within and across treatment arms and their relationship to starting physiologic and microbial community states. A weakness of the study is that it is not possible to attain full blinding since the test formulations are not packaged.

## Data Availability

Not applicable.

## Abbreviations

DNA: Deoxyribonucleic acid
ERC: Ethical Review Committee
LAZ: Length-for-age z-scores
MAM: Moderate Acute Malnutrition
MDCF: Microbiota Directed Complementary Food
MUAC: Mid upper arm circumference
POC: Proof-of-concept
RRC: Research Review Committee
RUSF: Ready to Use Supplementary Food
SAM: Severe Acute Malnutrition
WLZ: Weight-for-length z-scores
